# Schmerz und Schmerzmanagement in einem Pflegeheim: Ergebnisse einer Querschnittsstudie

**DOI:** 10.1007/s00482-025-00919-0

**Published:** 2026-01-14

**Authors:** Eva Pichler, Daniela Schoberer, Doris Eglseer, Wolfgang Strobl, Manuela Hödl

**Affiliations:** 1 Institut für Pflegewissenschaft, Medizinische Universität Graz, Neue Stiftingtalstraße 6/P06 – WEST, 8010 Graz, Österreich; 2 Pflegewohnhaus Graz St. Peter, Caritas, Hubertusstraße 6, 8042 Graz, Österreich

**Keywords:** Schmerz, Schmerzmanagement, Langzeitpflege, Pain, Pain management, Long-term care

## Abstract

**Hintergrund:**

Pflegeheimbewohner_innen leiden häufig unter schmerzverursachenden Erkrankungen. Gemäß dem biopsychosozialen Modell von Egle et al. können Schmerzen auf unterschiedlichen Dimensionen die Betroffenen beeinträchtigen. Jedoch werden bei der Behandlung die unterschiedlichen Schmerzdimensionen häufig nicht berücksichtigt, und in vielen Fällen werden Schmerzen seitens des Pflegepersonals nicht identifiziert. Diese Studie zielt darauf ab, die Schmerzprävalenz sowie die gesetzten Interventionen zur Schmerzreduktion in einem österreichischen Pflegeheim zu erheben.

**Methodik:**

Die deskriptive Querschnittsstudie wurde mittels einer Gelegenheitsstichprobe in einem österreichischen Pflegeheim durchgeführt. Der Schwerpunkt der Erhebung lag auf der Erfassung der Schmerzprävalenz, -intensität und -dauer sowie der durchgeführten Interventionen zur Schmerzreduktion.

**Ergebnisse:**

Von 121 Bewohner_innen nahmen 67 teil. 39 Bewohner_innen gaben an, unter Schmerzen zu leiden, wobei der Großteil von ihnen von chronischen Schmerzen betroffen war. Bei den betroffenen Bewohner_innen wurden insgesamt 64 Interventionen durchgeführt, wobei es sich hauptsächlich um pharmakologische handelte.

**Schlussfolgerung:**

Nichtpharmakologische Interventionen spielen in der Schmerzbehandlung eine wichtige Rolle, wurden jedoch selten vom Pflegepersonal eingesetzt. Die Ergebnisse dieser Erhebung wurden mit Pflegepersonen des Pflegeheims diskutiert, und basierend darauf wurde eine Übersicht zu nichtpharmakologischen Interventionen erstellt. Der sogenannte „Werkzeugkoffer“ ist frei verfügbar und soll Pflegepersonen dabei unterstützen, gemeinsam mit den Betroffenen die geeignete Intervention zur Schmerzreduktion auszuwählen.

**Graphic abstract:**

**Figure:**
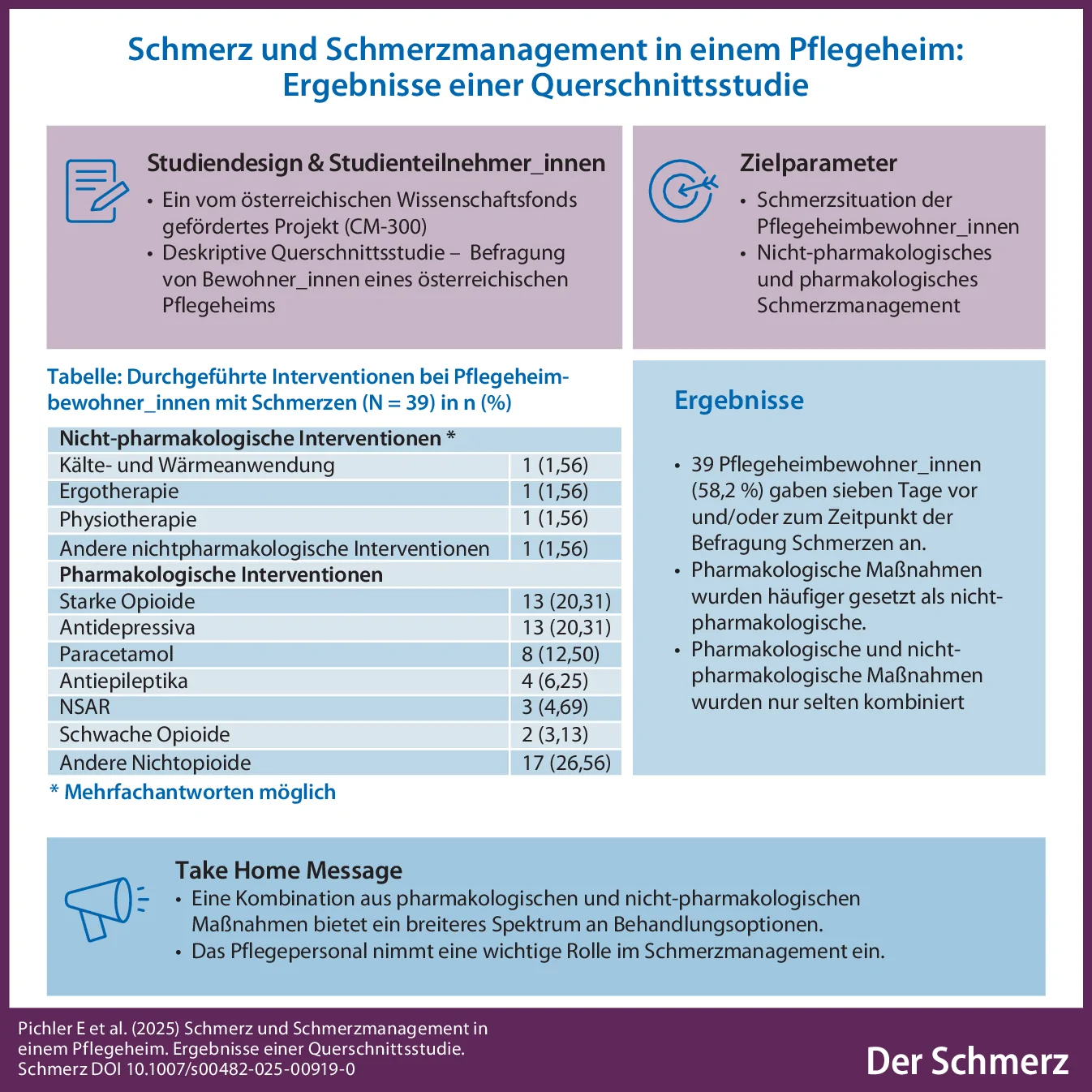

**Zusatzmaterial online:**

Die Online-Version dieses Beitrags (https://doi.org/10.1007/s00482-025-00919-0) enthält die STROBE-Statement-Checkliste.

## Abstract (en)

**Background:**

Nursing home residents frequently experience pain as a result of diseases. As posited by Egle et al., the bio-psycho-social model asserts that pain can affect individuals in various ways. Nevertheless, the various dimensions of pain are often not considered in pain management, and in numerous instances, pain is not even recognized by nursing professionals. The objective of the present study is to assess the prevalence of pain and the interventions implemented to reduce pain in an Austrian nursing home.

**Methods:**

For this descriptive cross-sectional study, convenience sampling in an Austrian nursing home was used. The focus of the data collection was to assess the prevalence, intensity, and duration of pain as well as the interventions used by the nurses to reduce pain.

**Results:**

Of 121 nursing home residents, 67 participated in the study: 39 residents had pain, whereby the majority had chronic pain. In total, 64 interventions were implemented, whereby most were pharmacological.

**Conclusion:**

Nonpharmacological interventions play an important role in pain management. However, they were seldom used by nursing staff. The results of the study were discussed with nursing staff at the nursing home and based on the discussion, a summary of nonpharmacological interventions was created. The so-called “toolbox” is freely available and is intended to support nursing staff in deciding with nursing home residents which interventions to use to reduce pain.

## Einblick in ein österreichisches Pflegeheim

Für eine Vielzahl der Pflegeheimbewohner_innen stellen Schmerzen ein alltägliches Problem dar. Pflegepersonen sehen sich mit der Herausforderung konfrontiert, Schmerzen zu identifizieren und adäquat zu reagieren. Obwohl in der wissenschaftlichen Literatur eine Kombination unterschiedlicher Interventionen zur Schmerzreduktion empfohlen wird, werden in der praktischen Anwendung Schmerzen meist sehr einseitig behandelt. Der vorliegende Beitrag offeriert einen Einblick in die Schmerzsituation sowie einen Überblick über die gesetzten Interventionen zur Schmerzreduktion in einem österreichischen Pflegeheim.

## Einleitung und Problemstellung

**Abb. 1:**
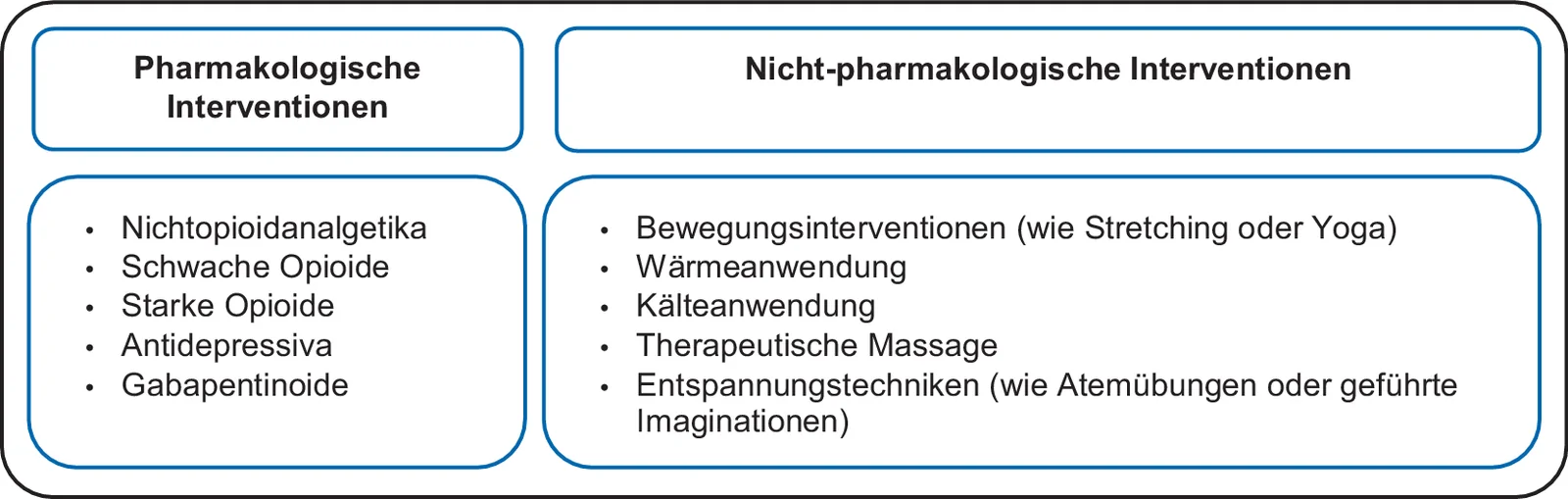
Beispielhafte Auflistung von pharmakologischen und nichtpharmakologischen Interventionen zur Schmerzreduktion bei älteren Menschen laut Levenson et al. 2021 [11]

Pflegeheimbewohner_innen sind meist von schmerzverursachenden (chronischen) Erkrankungen betroffen, was zu einer hohen Schmerzprävalenz bei dieser Personengruppe führt [1, 2]. In der internationalen Literatur wird ein Anteil von bis zu 85 % in Pflegeheimen angeführt [3–6]. Insbesondere chronische Schmerzen treten bei Personen über 65 Jahre sehr häufig auf [7–9]. In diesem Zusammenhang ist zu berücksichtigen, dass Schmerzen die Betroffenen nicht nur auf der physischen Ebene beeinflussen [10]. Das biopsychosoziale Modell von Egle et al. [10] zeigt die Multidimensionalität von Schmerz auf und gibt Betroffenen sowie dem Gesundheitspersonal einen Überblick über die verschiedenen Schmerzdimensionen und ihre komplexen Wechselwirkungen. Zu diesen Dimensionen gehören die biologische beziehungsweise physiologische (Gene, Erkrankungen oder der Gesundheitszustand), die soziale (das soziale Netzwerk, vergangene Schmerzerfahrungen oder die eigene Kultur) und die psychologische Dimension (Copingstrategien, Persönlichkeit oder Einstellungen; [10]). Um alle Dimensionen von Schmerz in der Behandlung berücksichtigen zu können, wird eine Kombination aus pharmakologischen und nichtpharmakologischen Interventionen empfohlen. Eine beispielhafte Auflistung der verschiedenen Interventionen zur Schmerzreduktion bei älteren Menschen kann Abb. 1 entnommen werden.

Darüber hinaus ist die Kooperation eines interdisziplinären Teams im Hinblick auf das Schmerzmanagement von zentraler Bedeutung. Das Team setzt sich in der Regel aus Pflegepersonen, Ärzt_innen, Physiotherapeut_innen und Psycholog_innen zusammen [11].

Im Rahmen der Schmerzbehandlung älterer Menschen sollte nicht nur auf die Reduktion der Intensität von Schmerzen gesetzt werden, sondern auch auf die Reduktion der Nebenwirkungen von pharmakologischen Interventionen [12]. Aufgrund von physiologischen, durch den Alterungsprozess bedingten Veränderungen können Nebenwirkungen stärker beeinträchtigen sowie häufiger auftreten. Weiters ist die Pharmakodynamik verändert; so werden Schmerzmedikamente schlechter verstoffwechselt, insbesondere wenn eine Mangelernährung vorherrscht. Daher sind eine angemessene Dosierung und die Vermeidung von Nebenwirkungen sowie von Über- und Unterbehandlung von besonderer Relevanz bei älteren Menschen [2].

Jedoch erfahren Pflegeheimbewohner_innen häufig nicht die erforderliche Behandlung, da Schmerzen in vielen Fällen vonseiten des Pflegepersonals nicht erkannt werden [13]. Darüber hinaus wird meist nur eine Schmerzdimension (wie die biologische/physiologische) durch die gesetzten Interventionen (wie Medikamente) adressiert. Dies führt dazu, dass andere Schmerzsymptome (wie psychische Belastung) nicht behandelt werden [14].

## Ziele

Die vorliegende Studie hat das Ziel, die Schmerzsituation der Pflegeheimbewohner_innen zu erfassen und das Schmerzmanagement in einem österreichischen Pflegeheim zu beschreiben. Dies soll dazu dienen, die Weiterentwicklung des Schmerzmanagements in diesem Setting zu ermöglichen.

## Methode

### Design

Diese deskriptive Querschnittsstudie wurde im Rahmen eines vom Österreichischen Wissenschaftsfonds geförderten Projekts (CM-300) durchgeführt. In diesem Projekt forcieren Pflegewissenschaftler_innen mit Pflegepraktiker_innen Wissensgenerierung und Forschungsimplementierung in einem österreichischen Pflegeheim. Für die Planung und Berichterstattung der Studie wurde die *Strengthening the Reporting of Observational Studies in Epidemiology Checklist* (STROBE; [15]) verwendet.

### Setting

Die Datenerhebung wurde in einem österreichischen Pflegeheim mit 116 Betten durchgeführt, welches nichtprofitorientiert ausgerichtet ist und sich in einem städtischen Gebiet befindet.

### Stichprobe

Insgesamt wurden im Erhebungszeitraum 121 Pflegeheimbewohner_innen vom Forschungsteam (zwei Pflegewissenschaftlerinnen und ein Diplom-Fachsozialbetreuer des Pflegeheims) zur Studienteilnahme eingeladen. Eine Stichprobengröße wurde im Vorhinein nicht berechnet, da für die Erhebung lediglich ein gewisser Zeitraum und ein Pflegeheim zur Verfügung standen. Alle Pflegeheimbewohner_innen, welche in diesem Zeitraum erreicht werden konnten, wurden für die Studienteilnahme in Betracht gezogen. Auch jene, die selbst nicht einwilligen können, wurden zur Datenerhebung eingeladen, sofern der_die Erwachsenenvertreter_in vorab eingewilligt hatte.

### Datensammlung

Die Datenerhebung wurde zwischen 1. Februar 2023 und 31. März 2023 in einem österreichischen Pflegeheim durchgeführt. Das Forschungsteam wurde bezüglich der Anwendung des Fragebogens trainiert, um ein standardisiertes Vorgehen gewährleisten zu können. Bei Pflegeheimbewohner_innen mit Erwachsenenvertretung wurde vor der Datensammlung durch das Forschungsteam in Kooperation mit den Pflegepersonen vor Ort evaluiert, ob die Durchführung eines Gesprächs zur Datenerhebung möglich war. Um fehlende Daten zu vermeiden, wurde der Fragebogen direkt nach der Datenerhebung durch eine zweite Person auf seine Vollständigkeit hin überprüft.

#### Instrument

Für die Datenerhebung wurde ein standardisierter Fragebogen verwendet, um Informationen über allgemeine Merkmale und Schmerzen der Pflegeheimbewohner_innen zu sammeln. Dieser aus mehreren Modulen bestehende Fragebogen wurde ursprünglich von einer internationalen Forschungsgruppe entwickelt. Sie besteht aus nationalen Koordinator_innen der teilnehmenden Länder, darunter die Niederlande, Österreich und die Schweiz [16]. Die Verwendung des Fragebogens wurde von den Entwickler_innen genehmigt. Das Modul zu den allgemeinen Merkmalen umfasst demografische Daten, das Eintrittsdatum ins Pflegeheim und medizinische Diagnosen gemäß ICD-11 (*International Statistical Classification of Diseases and Related Health Problems*; [17]). Zusätzlich wurde für diese Erhebung noch die Pflegestufe laut dem österreichischen System [18] mit aufgenommen. Das Schmerzmodul erfasst folgende Daten: (1) die in den letzten sieben Tagen empfundenen Schmerzen sowie deren Intensitätsgrad, (2) die zum Zeitpunkt der Messung empfundenen Schmerzen sowie deren Intensitätsgrad, (3) die Art der Schmerzen, die in den letzten sieben Tagen empfunden wurden, und (4) die zur Schmerzreduktion eingesetzten Interventionen. Für diese Studie wurden bei Punkt (2) Änderungen am ursprünglichen Fragebogen vorgenommen. Bei Pflegeheimbewohner_innen mit einem MMSE-2-Wert (*Mini-Mental Status Examination*) zwischen 17 und 0 wurde das Assessment-Tool *Pain Assessment in Impaired Cognition* (PAIC15) verwendet, da ein MMSE-2-Wert ab 17 auf eine moderate bis starke kognitive Beeinträchtigung hindeuten kann [19]. Je höher der Gesamtscore der PAIC15-Skala ist, desto wahrscheinlicher ist es, dass der_die Betroffene Schmerzen hat.

Dabei sind ab einem Grenzwert von 3 Schmerzen möglich und ab 4 wahrscheinlich [20]. Es gibt drei Kategorien: (1) Gesichtsausdruck, (2) Körperbewegung und (3) Vokalisation, mit je fünf Unterkategorien. Jede Unterkategorie kann mit „überhaupt nicht“ (0) bis „stark“ (3) bewertet werden. In jeder Kategorie können maximal 15 Punkte erreicht werden [21].

Zur Datensammlung bezüglich vergangener Schmerzereignisse und der derzeitigen Schmerzsituation wurden die Pflegeheimbewohner_innen selbst befragt. Bei einem vorliegenden MMSE-2-Wert von 17 bis 0 wurde zusätzlich die Pflegedokumentation hinzugezogen. Alle weiteren Daten wurden aus der Pflegedokumentation entnommen. Der gesamte Fragebogen findet sich in Tab. 1.

**Tab. 1:** Fragebogen zur Schmerzprävalenz sowie Informationen zur Datenquelle

Demografische Daten	Geburtsdatum	Pflegedokumentation
	Geschlecht	
	Aufnahmedatum	
	Pflegestufe	
Medizinische Diagnosen	☐ Bestimmte infektiöse und parasitäre Krankheiten ☐ Bösartige Neubildungen ☐ Krankheiten des Blutes und der blutbildenden Organe sowie bestimmte Störungen mit Beteiligung des Immunsystems ☐ Endokrine, Ernährungs- und Stoffwechselkrankheiten ☐ Psychische und Verhaltensstörungen ☐ Krankheiten des Nervensystems (ohne Verletzungen des Rückenmarks/Querschnittlähmung) ☐ Krankheiten des Auges und der Augenanhangsgebilde ☐ Krankheiten des Ohres und des Warzenfortsatzes ☐ Krankheiten des Kreislaufsystems ☐ Krankheiten des Atmungssystems ☐ Krankheiten des Verdauungssystems ☐ Krankheiten der Haut und der Unterhaut ☐ Krankheiten des Muskel-Skelett-Systems und des Bindegewebes ☐ Krankheiten des Urogenitalsystems ☐ Schwangerschaft, Geburt und Wochenbett ☐ Angeborene Fehlbildungen, Deformitäten und Chromosomenanomalien ☐ Symptome und abnorme klinische und Laborbefunde, die anderenorts nicht klassifiziert sind ☐ Verletzungen, Vergiftungen und bestimmte andere Folgen äußerer Ursachen ☐ Äußere Ursachen von Morbidität und Mortalität ☐ Faktoren, die den Gesundheitszustand beeinflussen und zur Inanspruchnahme des Gesundheitswesens führen ☐ Unbekannt/keine Diagnose	Pflegedokumentation
Schmerzprävalenz der letzten sieben Tage	Hat der/die Bewohner*in während der letzten sieben Tage unter Schmerzen gelitten? ☐ Keine Schmerzen ☐ Schmerzen vorhanden, aber nicht täglich ☐ Tägliche Schmerzen	Befragung des_der Bewohner_in Zusätzlich Pflegedokumentation bei einem MMSE-2-Wert von 17 bis 0
Durchschnittliche Schmerzintensität der letzten sieben Tage	Geben Sie die durchschnittliche Stärke der Schmerzen während der letzten sieben Tage an: ☐ Leichte Schmerzen ☐ Mäßige Schmerzen ☐ Starke Schmerzen ☐ Sehr starke Schmerzen ☐ Unerträgliche Schmerzen	Befragung des_der Bewohner_in Pflegedokumentation bei einem MMSE-2-Wert von 17 bis 0
Chronisch oder akut	Sind die Schmerzen chronisch oder akut? ☐ Chronisch ☐ Akut	Befragung des_der Bewohner_in Zusätzlich Pflegedokumentation bei einem MMSE-2-Wert von 17 bis 0
Schmerzprävalenz am Erhebungstag	Haben Sie derzeit Schmerzen ☐ Ja ☐ Nein	Befragung des_der Bewohner_in Zusätzlich Pflegedokumentation bei einem MMSE-2-Wert von 17 bis 0
Schmerzintensität am Erhebungstag	Bei Schmerzen: Geben Sie die Stärke der momentanen Schmerzen auf einer Skala von 1 bis 10 (NRS) an, wenn 10 der stärkste Schmerz ist. (Bei einem MMSE-2-Wert von 30 bis 18 Punkten) ☐ 1–10 Erhebung mittels PAIC (bei einem MMSE-2-Wert von 17 bis 0 Punkten)	NRS: Befragung des_der Bewohner_in PAIC: Proxy-Erhebung durch Pflegeperson
Schmerzinterventionen	☐ **Nicht-medikamentöse Maßnahmen** ☐ Physiotherapie ☐ Transkutane elektrische Nervenstimulation (TENS) ☐ Akupunktur ☐ Ruhigstellen ☐ Psychotherapie und Verhaltenstherapie ☐ Ergotherapie ☐ Musiktherapie ☐ Kälte- und Wärmetherapie ☐ Bewohner_innenedukation ☐ Entspannungstherapien (Yoga, Achtsamkeit etc.) ☐ Andere nichtmedikamentöse Maßnahmen ☐ **Medikamentöse Maßnahmen** ☐ *Nichtopioide* ☐ Paracetamol ☐ Nichtsteroidales Antirheumatikum (NSAR) ☐ Antidepressiva ☐ Antiepileptika (Gabapentin, Pregabalin etc.) ☐ Andere Nichtopioide ☐ *Opioide* ☐ Schwachwirksame Opioide (Codein oder Tramadol) ☐ Starke Opioide ☐ **Andere Maßnahmen** ☐ **Keine Maßnahmen** ☐ **Bewohner_in lehnt alle Maßnahmen zur Schmerzreduktion ab**	Pflegedokumentation

*NRS* „numeric rating scale“

### Datenanalyse

Die Daten wurden mit IBM SPSS Statistik Version 27 (IBM, Armonk, NY, USA; [22]) analysiert. Zur Beschreibung der Stichprobe wurden deskriptive Statistiken der Schmerzprävalenz, der Schmerzintensität und der vom Pflegepersonal durchgeführten Interventionen erstellt. Vor der Analyse wurden die Daten bereinigt und nur vollständige Datensätze wurden in die Analyse miteinbezogen. Ausgenommen hiervon waren die Angaben zur Pflegestufe.

### Ethische Aspekte

Die Studie wurde in Übereinstimmung mit der Erklärung von Helsinki und der „guten wissenschaftlichen Praxis“ durchgeführt. Ein Ethikvotum wurde vorab eingeholt (35-093 ex 22/23). Nach einer mündlichen Aufklärung gab jede_r teilnehmende Pflegeheimbewohner_in oder sein_ihre gesetzliche_r Vertreter_in vor der Datenerhebung eine schriftliche Einwilligung ab. Die Pflegeheimbewohner_innen hatten ausreichend Zeit, sich für oder gegen eine Teilnahme zu entscheiden. Ihnen wurde die Möglichkeit gegeben, Fragen zu stellen; flexible Pausen waren jederzeit möglich und die Datenerhebung fand an einem von den Pflegeheimbewohner_innen bevorzugten Ort statt. Weiters wurde den Pflegeheimbewohner_innen erklärt, dass die Teilnahme freiwillig ist, sich nicht auf die Betreuung und Pflege im Pflegeheim auswirkt und sie jederzeit die Erhebung abbrechen oder von der Studienteilnahme zurücktreten können.

## Ergebnisse

### Demografische Daten

Von insgesamt 121 Pflegeheimbewohner_innen nahmen 67 (55,4 %) an der Erhebung teil. Die 67 teilnehmenden Pflegeheimbewohner_innen waren überwiegend weiblich (73,1 %) und ihr mittleres Alter betrug 84 Jahre (IQB 12). Die Beschreibung der Stichprobe findet sich in Tab. 2.

**Tab. 2:** Beschreibung der Stichprobe

*Grund für Nichtteilnahme, n (%)*	*(n* *=* *54)*
Kein Grund	34 (62,96)
Teilnahme nicht möglich (z. B. schlechter Gesundheitszustand)	10 (18,52)
Pflegeheimbewohner_in während Erhebungsperiode verstorben	2 (3,70)
Pflegeheimbewohner_in während Erhebungsperiode verzogen	2 (3,70)
Erwachsenenvertretung nicht erreicht	6 (11,11)
*Teilnehmende Pflegeheimbewohner_innen*	*(n* *=* *67)*
*Alter Median (IQB)*	84 (12)
*Geschlecht, n (%)*	
Weiblich	49 (73,13)
*Aufenthaltsdauer in Monaten, Median (IQB)*	20 (32)
Pflegestufe, n (%)*	
Pflegestufe 2	3 (4,69)
Pflegestufe 3	12 (18,75)
Pflegestufe 4	24 (37,50)
Pflegestufe 5	22 (34,38)
Pflegestufe 6	3 (4,76)
*Häufigste Diagnosen in n (%)*^*+*^	
Krankheiten des Kreislaufsystems	53 (79,10)
Geistes‑/Verhaltensstörungen (ohne Demenz, durch psychotrope Substanzen verursachte Störungen und Sucht)	29 (43,28)
Erkrankungen des Muskel-Skelett-Systems und des Bindegewebes	29 (43,28)
Erkrankungen des Hormonsystems, der Ernährung und des Stoffwechsels (ohne Diabetes mellitus)	27 (40,83)
*Anzahl der Diagnosen pro Person, Median (IQB)*	5 (3)

*Bei drei Pflegeheimbewohner_innen war zum Zeitpunkt der Befragung keine Pflegestufeneinschätzung dokumentiert

^+^Einige Pflegeheimbewohner_innen hatten mehrere Krankheiten innerhalb einer Kategorie. Diese wurden nicht einzeln, sondern als eine Diagnose gezählt

*IQB* Interquartilsbereich

## Schmerz

Insgesamt gaben 39 Pflegeheimbewohner_innen (58,2 %) sieben Tage vor und/oder zum Zeitpunkt der Befragung Schmerzen an. 81,6 % der Pflegeheimbewohner_innen, die in den letzten sieben Tagen an Schmerzen gelitten hatten, gaben chronische Schmerzen an. Die meisten von ihnen (60,5 %) stuften ihre Schmerzen als mäßig ein. Der Median der Numeric-rating-scale(NRS)-Werte lag bei 5 (IQB 2). Bei zwei Pflegeheimbewohner_innen wurde der Schmerz mit der PAIC15 eingeschätzt; ein Gesamtscore lag bei acht, was auf wahrscheinliche Schmerzen hinweist, und einer bei zwei, was darauf hindeutet, dass keine Schmerzen vorlagen. Die Ergebnisse der Schmerzerhebung sind in Tab. 3 dargestellt.

**Tab. 3:** Schmerzprävalenz und Schmerzmerkmale der teilnehmenden Pflegeheimbewohner_innen (*N* = 67) in *n* (%)

**Schmerzen in den letzten sieben Tagen und zum Zeitpunkt der Befragung, *n*** **(%)**	
Ja	39 (58,20)
**Schmerzen in den letzten sieben Tagen, *n*** **(%)**	
Keine	29 (43,28)
Schmerzen, aber nicht täglich	18 (26,87)
Tägliche Schmerzen	20 (29,85)
*Schmerzintensität, n (%)*	
Leichte	6 (15,79)
Mäßige	23 (60,53)
Starke	8 (21,05)
Sehr starke	1 (2,63)
*Schmerzart, n (%)*	
Chronisch	31 (81,58)
**Schmerzen zum Zeitpunkt der Befragung, *n*** **(%)**	
Ja	20 (29,85)
*Stärke der Schmerzen zum Zeitpunkt der Befragung, gemessen mit NRS (N* *=* *18), n (%)*	
2	1 (5,56)
3	1 (5,56)
4	1 (5,56)
5	8 (44,44)
6	2 (11,11)
7	2 (11,11)
8	3 (16,67)
Median (IQB)	5 (2)
*PAIC15-Gesamtscore (N* *=* *2), n (%)*	
2	1 (50)
8	1 (50)

## Interventionen zur Schmerzreduktion

**Abb. 2:**
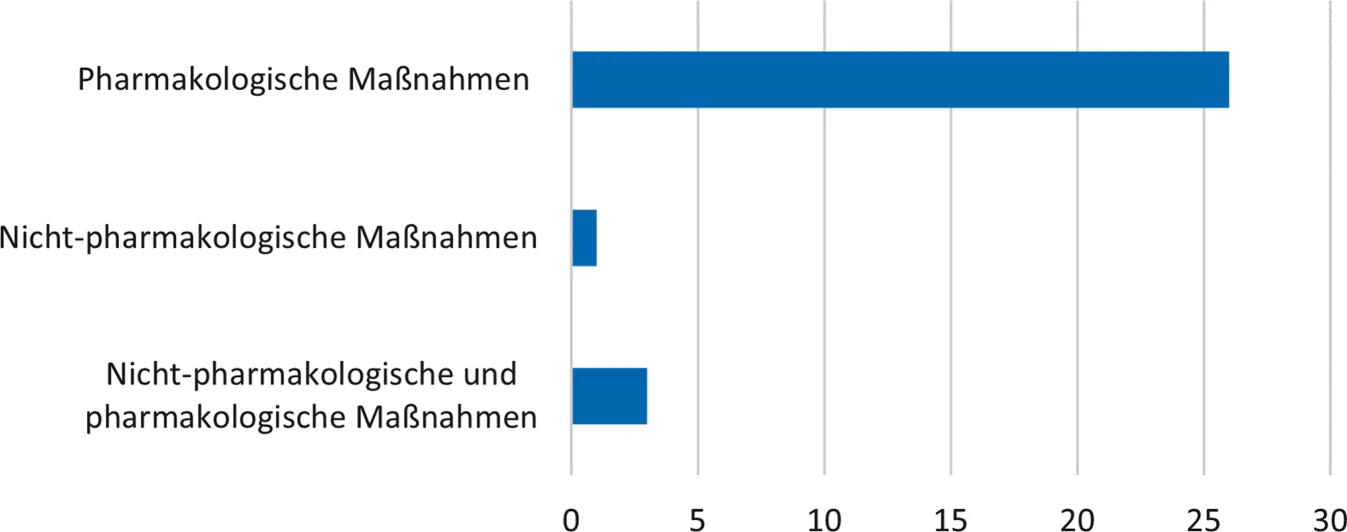
Anzahl der Personen, die pharmakologische Maßnahmen, nichtpharmakologische Maßnahmen oder beides erhielten, in absoluten Zahlen (*N* = 39)

**Abb. 3:**
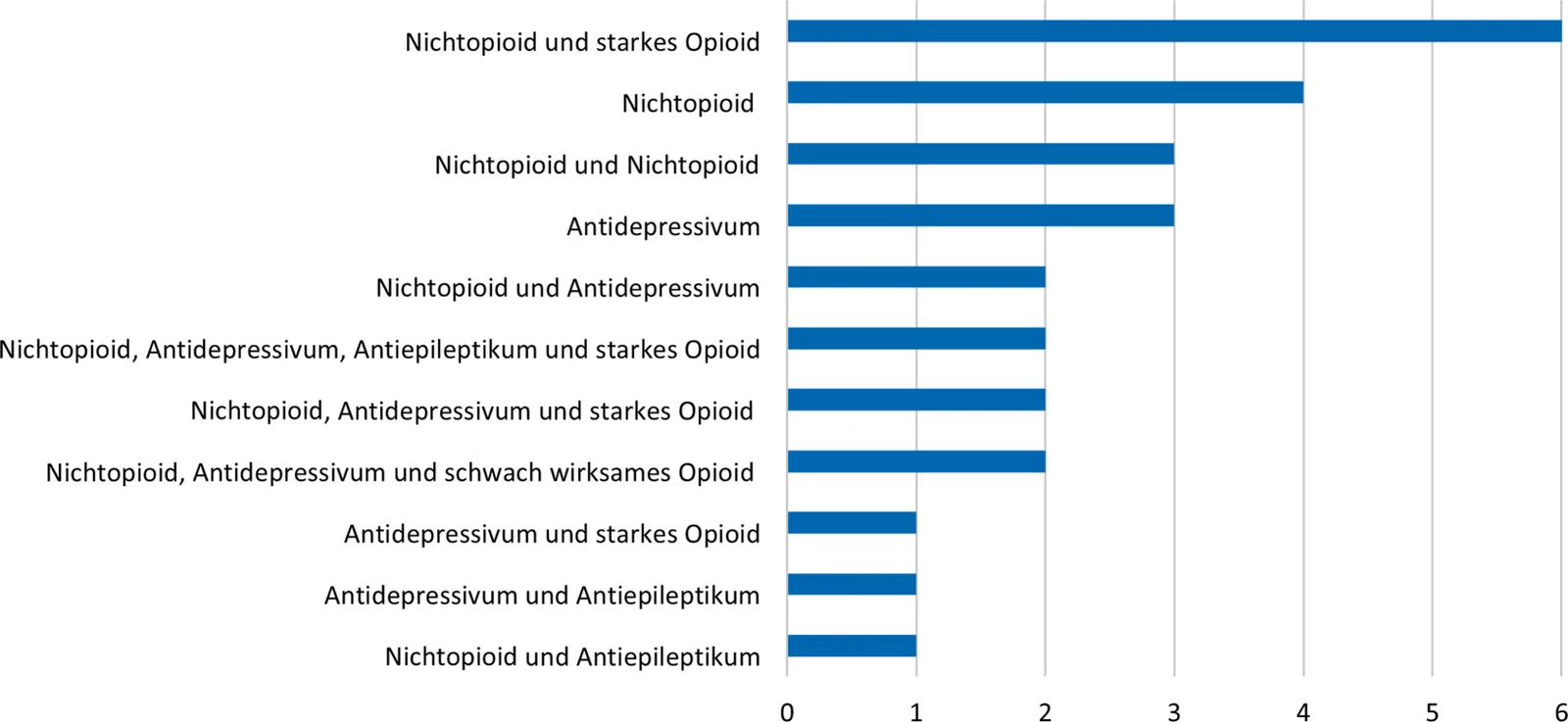
Kombination der Medikamente in absoluten Zahlen (*N* = 29)

Insgesamt wurden bei 39 (58,2 %) Pflegeheimbewohner_innen mit Schmerzen 64 Interventionen vom Pflegepersonal durchgeführt. Pharmakologische Interventionen wurden häufiger durchgeführt als nichtpharmakologische (Tab. 4). 29 (74,4 %) der 39 (100 %) Pflegeheimbewohner_innen mit Schmerzen erhielten Medikamente, welche nur in seltenen Fällen mit nichtpharmakologischen Maßnahmen kombiniert wurden (siehe Abb. 2). Am häufigsten wurden Nichtopioide mit einem starken Opioid kombiniert; weitere Kombinationen können Abb. 3 entnommen werden. Bei zehn (25,6 %) Pflegeheimbewohner_innen wurde ein Schmerzassessment durchgeführt, und neun (23,1 %) haben trotz Schmerzen keine Interventionen erhalten. Diese Pflegeheimbewohner_innen gehörten auch zu jenen, bei denen kein Schmerzassessment durchgeführt wurde.

**Tab. 4:** Durchgeführte Interventionen bei Pflegeheimbewohner_innen mit Schmerzen (*N* = 39) in *n* (%)

Nichtpharmakologische Interventionen*	
Kälte- und Wärmeanwendung	1 (1,56)
Ergotherapie	1 (1,56)
Physiotherapie	1 (1,56)
Andere nichtpharmakologische Interventionen	1 (1,56)
*Pharmakologische Interventionen*	
Starke Opioide	13 (20,31)
Antidepressiva	13 (20,31)
Paracetamol	8 (12,50)
Antiepileptika	4 (6,25)
NSAR	3 (4,69)
Schwache Opioide	2 (3,13)
Andere Nichtopioide	17 (26,56)

*Mehrfachantworten möglich

## Diskussion

Die vorliegende Studie zielte darauf ab, die Schmerzprävalenz von Pflegeheimbewohner_innen in einem österreichischen Pflegeheim sowie die gesetzten Interventionen zur Schmerzreduktion zu erheben. An der Erhebung nahmen 67 Pflegeheimbewohner_innen teil, von denen mehr als 50 % angaben, innerhalb der letzten sieben Tage und/oder zum Zeitpunkt der Befragung Schmerzen gehabt zu haben. Basierend auf internationalen Studien kann in Pflegeheimen eine Schmerzprävalenz mit der Spanne von 55 bis 85 % angenommen werden [3–5]. Die hier erhobene Schmerzprävalenz liegt somit mit 58,2 % im unteren Bereich. Im Hinblick auf den Vergleich von Schmerzprävalenzen mit internationaler Literatur ist zu berücksichtigen, dass die einzelnen Studien unterschiedliche Ziele verfolgen und die Methodik der Schmerzerhebung sich unterscheidet. Das Ergebnis einer Schmerzerhebung wird maßgeblich durch die gewählte Zeitspanne der Schmerzerfassung, die Quelle der Schmerzdaten, das Assessment-Tool und die Charakteristika der Betroffenen beeinflusst [3].

Für chronische Schmerzen in Pflegeheimen liegt die Prävalenz laut der internationalen Literatur zwischen 29,3 und 78 % [3, 23, 24]. Mit 81,6 % liegt der Wert der vorliegenden Studie über dem internationalen Bereich. Hier gilt es zu beachten, dass sich die Einordnung der Schmerzen in chronisch oder akut nach Selbstauskunft und nicht nach einer medizinischen Diagnose richtete.

Die überwiegende Anzahl der Pflegeheimbewohner_innen wurde mit pharmakologischen Interventionen behandelt, wohingegen nichtpharmakologische Interventionen nur in begrenztem Umfang durchgeführt wurden. In den seltenen Fällen ihrer Anwendung wurden diese hauptsächlich in Kombination mit pharmakologischen Interventionen eingesetzt. Die Kombination aus unterschiedlichen Interventionen sollte immer forciert werden, da diese Behandlung wirksamer ist und zu einer langfristigeren Schmerzreduktion führt als der Einsatz von einzelnen Interventionen. Zudem kann durch eine Kombination aus pharmakologischen und nichtpharmakologischen Interventionen den Betroffenen ein breiteres Spektrum an Behandlungsoptionen angeboten werden [11]. Insbesondere bei älteren Personen empfiehlt sich, unterschiedliche Behandlungsansätze zu nutzen. Der ausschließliche Fokus auf pharmakologische Interventionen kann das Risiko für Polymedikation und Nebenwirkungen erhöhen [25]. Zudem führen pharmakologische Interventionen bei älteren Personen häufig zu Nebenwirkungen wie Harnverhalt, Verstopfung, Sedierung und einem damit verbundenen erhöhten Sturzrisiko [8].

Die Resultate stehen im Konsens mit anderen Studien, die darauf hindeuten, dass nichtpharmakologische Interventionen in der täglichen Routine seltener eingesetzt werden als pharmakologische [3, 26, 27]. Die vorliegenden Daten ermöglichen keine Ableitung der Frage, warum in diesem Pflegeheim fast ausschließlich pharmakologische Interventionen zum Einsatz kamen. Entscheidende Faktoren bezüglich des Einsatzes von nichtpharmakologischen Interventionen sind laut Literatur der Ausbildungsgrad, die Qualifikation und das Training von Pflegepersonen. Pflegepersonen mit niedriger Qualifikation und wenig Training im Schmerzmanagement sind meist unsicher im Umgang mit den unterschiedlichen Behandlungsoptionen, wodurch nichtpharmakologische Interventionen seltener gesetzt werden [28, 29]. Ob diese Erklärung bei der vorliegenden Erhebung ebenfalls Gültigkeit besitzt, kann nicht mit Sicherheit festgestellt werden. Fest steht aber, dass in Österreich überwiegend niedriger qualifiziertes Personal in Pflegeheimen tätig ist [30]. Internationale Studien zeigen zudem, dass es in Pflegeheimen sehr häufig an Schmerzfortbildungen mangelt [29, 31].

Neun der 39 Pflegeheimbewohner_innen erhielten trotz Schmerzen keine Interventionen. Wieso dies so war, lässt sich anhand der Daten nicht aufzeigen. Es ist bereits bekannt, dass Schmerzen vor allem bei älteren Menschen häufig nicht bemerkt und somit nicht behandelt werden. Dabei spielt das Schmerzassessment eine wichtige Rolle. Häufig wird dieses durch kognitive Beeinträchtigungen, sensorische Schwierigkeiten oder Kommunikationsprobleme erschwert [13]. Auch in der vorliegenden Erhebung zeigte sich, dass Schmerzassessments insgesamt nur selten durchgeführt wurden, insbesondere bei jenen Pflegeheimbewohner_innen, die keine Interventionen erhalten haben. Die oben genannten Schwierigkeiten bei der Durchführung eines Schmerzassessments trafen (zumindest während des Erhebungszeitraums) auf die teilnehmenden Pflegeheimbewohner_innen nicht zu. Ong und Thiam [13] berichteten von einem weiteren Faktor, der das Schmerzassessment beeinflusst: So tendieren nicht alle Betroffenen bei Schmerzen gleichermaßen dazu, Hilfe zu suchen. Für die vorliegende Erhebung ist es allerdings retrospektiv schwierig nachzuvollziehen, ob dieser Faktor beim Schmerzassessment ebenfalls als hinderlich hinzukam.

### Limitationen

Bei der Interpretation der vorliegenden Studie sind einige Limitationen zu berücksichtigen. Die Anzahl der Teilnehmer_innen war vergleichsweise gering, und es wurde nur ein österreichisches Pflegeheim untersucht, weshalb eine Generalisierbarkeit der vorliegenden Daten nicht möglich ist. Sie geben jedoch einen guten ersten Einblick in ein österreichisches Pflegeheim. Im Zusammenhang mit Fragen nach vergangenen Schmerzereignissen sollte immer erwähnt werden, dass diese Aussagen anfällig für Verzerrungen sind. Daher wurde nicht nur die bisherige Schmerzsituation erhoben, sondern auch die gegenwärtige. Die Nutzung von Fremdeinschätzungsinstrumenten ist ebenfalls mit Verzerrungen verbunden. Die Beobachtung und Beurteilung der Schmerzsituation durch eine andere Person (Pflegeperson) kann zu einer Über- oder Unterschätzung des Schmerzes führen. Dadurch könnten die Schmerzsituationen bei den zwei Pflegeheimbewohner_innen, welche mit einem Fremdeinschätzungsinstrument eingestuft wurden, falsch eingeschätzt worden sein.

Eine weitere Limitation ist mit der Verwendung des Fragebogens verbunden. Die Originalversion wurde psychometrisch getestet; für die deutsche Version liegen allerdings keine Daten zur psychometrischen Evaluierung vor. Weiters wurde der Fragebogen in gewissen Punkten für die vorliegende Studie adaptiert und entspricht somit nicht mehr vollständig dem ursprünglichen Fragebogen.

## Schlussfolgerung

In dieser Studie konnte gezeigt werden, dass in einem österreichischen Pflegeheim nichtpharmakologische Interventionen zur Schmerzreduktion seltener von der Pflege durchgeführt wurden als pharmakologische. Assessments wurden nicht bei allen Pflegeheimbewohner_innen mit Schmerzen durchgeführt, und einige erhielten trotz Schmerzen keine Interventionen. Es ist wichtig, dass das Pflegepersonal ein Bewusstsein für das Vorhandensein von Schmerzen hat und dass die Betroffenen ein individuelles Schmerzmanagement erhalten. Durch die Präsenz im Alltag der betroffenen Pflegeheimbewohner_innen nimmt das Pflegepersonal eine wichtige Rolle im Erkennen, Behandeln und im Management von Schmerzen ein.

Basierend auf der Diskussion dieser Ergebnisse mit Pflegepersonen des Pflegeheims wurde eine Übersicht zu nichtpharmakologischen Interventionen erstellt. Der sogenannte „Werkzeugkoffer“ ist frei verfügbar und soll Pflegepersonen unter anderem dabei unterstützen, gemeinsam mit den Betroffenen geeignete nichtpharmakologische Interventionen zur Schmerzreduktion auszuwählen.

## Fazit für die Praxis

- Schmerz wird von den Betroffenen auf mehreren Dimensionen erlebt, daher sollten die Interventionen zur Schmerzreduktion diesem Erleben angepasst werden.
- Trotz der Empfehlungen aus der Literatur werden in der Praxis meist pharmakologische Interventionen zur Schmerzreduktion gesetzt.

## Supplementary Information

STROBE-Statement-Checkliste

## Data Availability

Aufgrund des Vertrags mit dem FWF dürfen die gesammelten Daten nicht an Dritte weitergegeben werden.
